# Crystallization and preliminary X-ray diffraction analysis of the complex between a human anti-interferon antibody fragment and human interferon α-2A

**DOI:** 10.1107/S1744309108037925

**Published:** 2008-12-25

**Authors:** Vaheh Oganesyan, Melissa M. Damschroder, Kimberly E. Cook, Herren Wu, William F. Dall’Acqua

**Affiliations:** a Department of Antibody Discovery and Protein Engineering, MedImmune, One MedImmune Way, Gaithersburg, MD 20878, USA

**Keywords:** interferon α-2A, antibodies, Fab fragments

## Abstract

Recombinant human interferon α-2A (rhIFN-α-2A) has been crystallized in complex with the recombinantly produced Fab fragment of a therapeutic monoclonal antibody (MEDI545; IgG1/κ) which targets several human inter­feron α subtypes. This constitutes the first reported crystals of a human type I interferon bound to an antibody. The orthorhombic crystals belonged to either space group *I*222 or *I*2_1_2_1_2_1_, with unit-cell parameters *a* = 134.82, *b* = 153.26, *c* = 163.49 Å. The diffraction of the crystals extended to 3.0 Å resolution. The asymmetric unit contained two Fab–rhIFN-α-2A complexes. This corresponded to a crystal volume per protein weight (*V*_M_) of 3.02 Å^3^ Da^−1^ and a solvent content of 59.3%. The corresponding three-dimensional structure is expected to shed light on the mechanism of action of MEDI545 and the molecular basis of its specificity.

## Abstract 2

Crystals of the complex between the Fab fragment of a human anti-interferon α therapeutic antibody and human interferon α-2A have been obtained and diffracted to 3.0 Å resolution.

## 1. Introduction

Type I interferons (IFNs) are a family of cytokines that are expressed from over 15 functional genes and include IFN-α, IFN-β, IFN-τ, IFN-­κ, IFN-∊, IFN-δ and IFN-ω. Interferons, in particular IFN-α and IFN-β, have historically been characterized in terms of their role in modulating the responses of mammalian hosts to infections (Isaacs & Lindenmann, 1957 ▶; Gresser *et al.*, 1976 ▶; Alsharifi *et al.*, 2008 ▶). How­ever, it is becoming increasingly clear that they also play a key role in immune regulation and autoimmune diseases (Baccala *et al.*, 2005 ▶; Theofilopoulos *et al.*, 2005 ▶). The IFN-α family alone comprises at least 13 different subtypes (Foster & Finter, 1998 ▶) and is thought to play a role in systemic lupus erythematosus (SLE), type I diabetes, Sjögren’s syndrome and thyroid diseases (Selmi *et al.*, 2006 ▶). This attractive targeting potential led to the development by MedImmune of MEDI545, a fully human monoclonal antibody which exhibits binding to multiple IFN-α subtypes. MEDI545 is currently undergoing several human clinical trials for various indications such as lupus, chronic plaque psoriasis, dermatomyositis and poliomyelitis (http://www.clinicaltrials.gov).

In an effort to better understand the molecular basis of the recognition of IFN-α molecules by MEDI545, we set out to solve the X-ray crystal structure of the complex between the Fab fragment of this antibody and human IFN-α-2A. Three type I human interferon structures have previously been determined using either X-ray crystallography or NMR. These correspond to human IFN-α-2a, IFN-­α-2b and IFN-β (Radhakrishnan *et al.*, 1996 ▶; Karpusas *et al.*, 1997 ▶; Klaus *et al.*, 1997 ▶). Here, the first reported diffracting crystals of a human type I interferon bound to an antibody are described and characterized.

## 2. Materials and methods

### 2.1. Expression and purification of MEDI545 Fab and rhIFN-α-2a

The heavy and light chains of the Fab fragment of MEDI545 (IgG1/κ) were cloned into a mammalian expression vector. More precisely, each chain was placed under the control of its own human cytomegalovirus major immediate early (hCMVie) promoter and enhancer (described in Boshart *et al.*, 1985 ▶). Each of those two genes also incorporated an SV40 poly-A sequence to allow proper pro­cessing of its 3′ end. In this system, a human γ1 chain truncated at position Lys235 (according to the Kabat numbering convention; Kabat *et al.*, 1991 ▶) is secreted along with a human κ chain (Oganesyan *et al.*, 2008 ▶). The construct was then transiently transfected into human embryonic kidney (HEK) 293 cells using Lipofectamine (Invitrogen, Carlsbad, California, USA) and standard protocols. MEDI545 Fab was typically harvested 72, 144 and 216 h post-transfection. Conditioned media containing the soluble Fab fragment were then dialyzed against 50 m*M* sodium acetate pH 5.2, 20 m*M* NaCl and purified using a HiTrap HP SP column in a NaCl gradient according to the manufacturer’s instructions (GE Healthcare, Piscataway, New Jersey, USA). Purified MEDI545 Fab, typically >95% homogeneous as judged by SDS–PAGE, was then concentrated to approximately 30 mg ml^−1^.

High-quality nearly 100% homogeneous *Escherichia coli*-expressed recombinant human interferon α-2A (rhIFN-α-2A) was purchased directly from Potomac Affinity Proteins LLC (Rockville, Maryland, USA). The cytokine’s mature sequence corresponded to residues 24–188 of entry No. P01563 of the Swiss-Prot protein database (http://www.expasy.org) and included two extra threonine and serine residues on its N-terminal end. MEDI545 Fab and rhIFN-α-2A were mixed in a 1.2:1 molar ratio. The resulting mixture was then adjusted to approximately 10 mg ml^−1^. The complex was further purified by size-exclusion chromatography using a Superdex S200 column (GE Healthcare) equilibrated with 20 m*M* sodium acetate pH 5.2, 20 m*M* NaCl at a flow rate of 0.5 ml min^−1^. After separation from excess MEDI545 Fab, the complex was concentrated to 6.5 mg ml^−1^ using an Amicon YM-30 concentrator (Millipore, Billerica, Massachusetts, USA).

### 2.2. Crystallization screening and optimization

Sitting-drop crystallization experiments were initially set up in 96-­well plates with conical flat-bottomed drop compartments (Corning 3785; VWR, West Chester, Pennsylvania, USA) using a Phoenix crystallization robot (Art Robbins, Sunnyvale, California, USA). Favorable conditions were initially identified using the following commercially available crystallization screens: Crystal Screen HT, Index Screen, SaltRx (Hampton Research, Aliso Viejo, California, USA) and ProPlex (Molecular Dimensions, Apopka, Florida, USA). For further optimization purposes, a commercially available kit was used (Additive Screen HT; Hampton Research). More particularly, in screening mode the reservoirs and drop compartments of the 96-well plates were filled with 99 and 0.3 µl, respectively, of the various screen solutions using the Phoenix robot. 0.3 µl of the MEDI545 Fab–rhIFN-α-2A complex at a concentration of 6.5 mg ml^−1^ was then added to the drop compartment. In optimization mode, the reservoirs were first filled with 80 µl screen solution. 20 µl of the various additives were then added and the reservoirs were subjected to five cycles of mixing by aspiration/dispensing. 0.3 µl of each reservoir solution was then mixed with 0.3 µl of the MEDI545 Fab–rhIFN-α-2A complex at a concentration of 6.5 mg ml^−1^ in the drop compartment. Diffraction-quality crystals were grown in hanging drops once optimal conditions had been identified (see §[Sec sec3]3).

### 2.3. X-ray diffraction data collection and processing

Diffraction data were collected from a single crystal at the Center for Advanced Research in Biotechnology (CARB, University of Maryland Biotechnology Institute, Rockville, Maryland, USA) using a MicroMax-007 rotating-anode generator fitted with an R-AXIS IV^++^ imaging plate (Rigaku/MSC, The Woodlands, Texas, USA). The crystal was harvested using a litho-loop and flash-cooled in a liquid-nitrogen stream using an X-stream 2000 cryogenic cooler (Rigaku/MSC). Prior to this step, cryoprotection was achieved by soaking the crystal in 200 m*M* NaI, 8 m*M* NiCl_2_.6H_2_O, 80 m*M* Tris–HCl pH 8.5, 16%(*w*/*v*) polyethylene glycol monomethyl ether 2000, 20% glycerol. 240 consecutive images were collected using an oscillation range of 0.5°, a crystal-to-detector distance of 250 mm and an exposure time of 600 s. The diffraction images were integrated and scaled using *HKL*-2000 (Otwinowski & Minor, 1997 ▶).

## 3. Results and discussion

**Figure 1:**
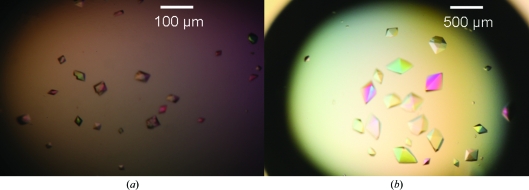
Crystals of the MEDI545 Fab–rhIFN-α-2a complex. (*a*) Screening. The crystals shown were obtained during the initial screening phase using Crystal Screen HT (Hampton Research) under condition H9 [0.01 *M* NiCl_2_.6H_2_O, 0.1 *M* Tris–HCl pH 8.5, 20%(*w*/*v*) polyethylene glycol monomethyl ether 2000]. (*b*) Optimization. The crystals shown grew to dimensions of up to 0.1 × 0.1 × 0.2 mm in hanging drops when 1 µl reservoir solution [200 m*M* NaI, 8 m*M* NiCl_2_.6H_2_O, 80 m*M* Tris–HCl pH 8.5, 16%(*w*/*v*) polyethylene glycol monomethyl ether 2000] was mixed with 1 µl of a 3.5 mg ml^−1^ MEDI545 Fab–rhIFN-α-2A solution in 20 m*M* sodium acetate pH 5.2, 20 m*M* NaCl.

Crystals of less than 10 µm in size and of tetragonal bipyramidal shape appeared during the screening process with Crystal Screen HT under condition H9 [0.01 *M* NiCl_2_.6H_2_O, 0.1 *M* Tris–HCl pH 8.5, 20%(*w*/*v*) polyethylene glycol monomethyl ether 2000] as seen in Fig. 1 ▶(*a*). During the optimization process, crystals of similar morphology appeared in almost all of the conditions of the Additive Screen. However, the largest crystals were obtained when using additives B8 (1.0 *M* NaI), D3 (0.1 *M* spermine–HCl) and D4 [0.1 *M* cobalt(III) hexammine chloride]. Hanging drops were then set up under those various conditions using 1 µl of the MEDI545 Fab–rhIFN-α-2A complex at a concentration of 3.5 mg ml^−1^ in 20 m*M* sodium acetate pH 5.2, 20 m*M* NaCl and 1 µl of the appropriate reservoir solutions. Diffraction-quality orthorhombic crystals grew in about 15 d when 200 m*M* NaI, 8 m*M* NiCl_2_.6H_2_O, 80 m*M* Tris–HCl pH 8.5, 16%(*w*/*v*) polyethylene glycol monomethyl ether 2000 was used as the reservoir solution. They reached dimensions of up to 0.1 × 0.1 × 0.2 mm, as seen in Fig. 1 ▶(*b*).

**Figure 2:**
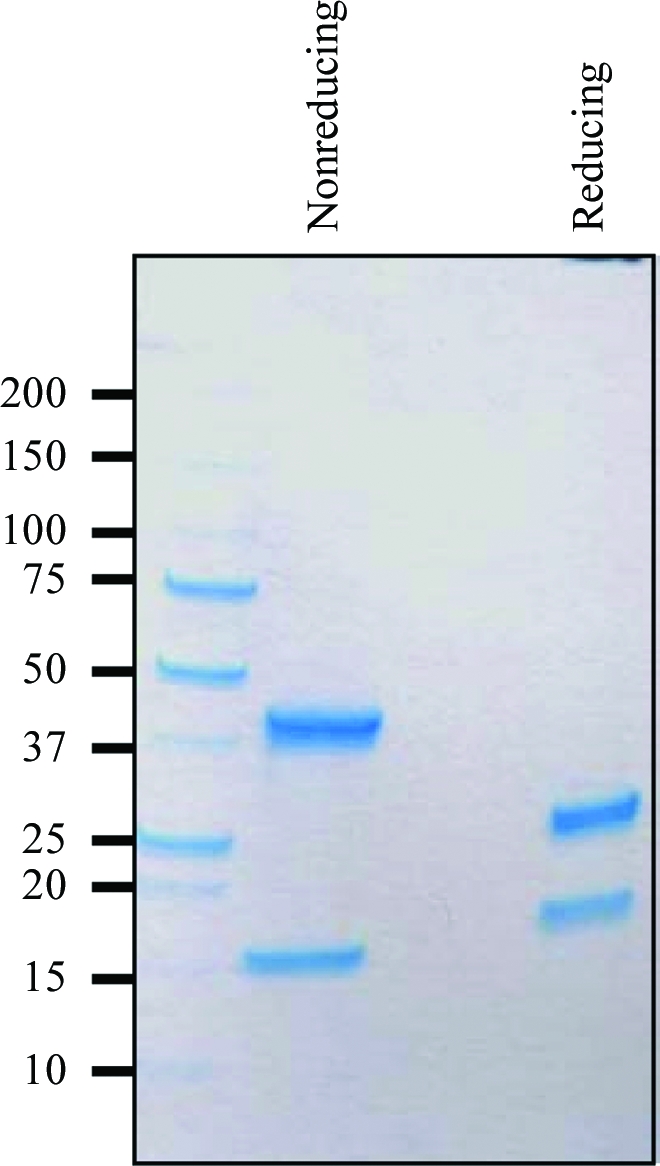
SDS–PAGE profile of a dissolved crystal of the MEDI545 Fab–rhIFN-α-2a complex under reducing and nonreducing conditions. The 4–20% gradient gel (BioRad, Hercules, California, USA) was stained with Bio-Safe Coomassie Stain (BioRad). It shows rhIFN-α-2a migrating at around 15 and 20 kDa under nonreducing and reducing conditions, respectively. This difference is likely to be attributable to the presence of two internal disulfide bonds, the reduction of which alters the compactness (and thus the migration) of the molecule. The same argument can be applied to the MEDI545 Fab fragment, which migrated slightly under its expected molecular weight of 50 kDa under nonreducing conditions. As expected, its heavy and light chains both migrated at 25 kDa under reducing conditions.

SDS–PAGE analysis of these crystals confirmed that they indeed contained the expected complex formed by MEDI545 Fab and the recombinant human interferon α-2A (see Fig. 2 ▶). Incidentally, the SDS–PAGE profile of MEDI545 Fab also demonstrated the presence of the expected interchain disulfide bond between Cκ/Cys214 and C_H_1/Cys233 (Kabat numbering; Kabat *et al.*, 1991 ▶) owing to its differential migration pattern under reducing and nonreducing con­ditions (see Fig. 2 ▶).

**Figure 3:**
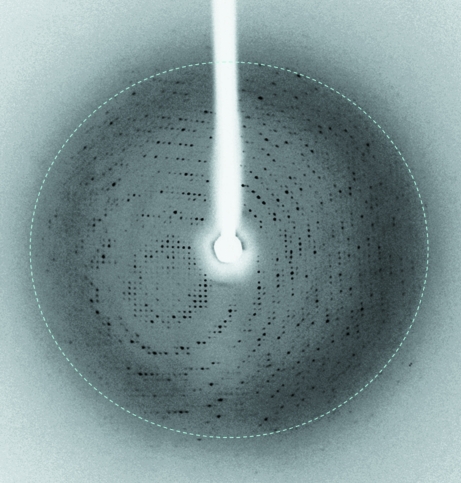
Diffraction image of a MEDI545 Fab–rhIFN-α-2a crystal.

The diffraction limit of the crystal was 3.0 Å (see Fig. 3 ▶). The possible space groups were determined to be either *I*222 or *I*2_1_2_1_2_1_, with unit-cell parameters *a* = 134.82, *b* = 153.26, *c* = 163.49 Å. The asymmetric unit contained two MEDI545 Fab–rhIFN-α-2A com­plexes. This corresponded to a *V*_M_ of 3.02 Å^3^ Da^−1^ and a solvent content of 59.3%. Data-collection statistics are shown in Table 1 ▶. Structure determination using molecular replacement is currently under way.

## Figures and Tables

**Table 1:** X-ray diffraction data-collection statistics Values in parentheses are for the highest resolution shell.

Wavelength (Å)	1.54
Resolution (Å)	23.0–3.0 (3.11–3.0)
Space group	*I*222 or *I*2_1_2_1_2_1_
Unit-cell parameters (Å)	*a* = 134.82, *b* = 153.26, *c* = 163.49
Total reflections	156727
Unique reflections	34084
Average redundancy	4.6 (4.3)
Completeness (%)	99.8 (99.4)
*R*_merge_	0.119 (0.423)
Mean *I*/σ(*I*)	7.8 (2.7)
